# Multinomial Estimations of Predictive Risk Factors for Traumatic Brain Injuries

**DOI:** 10.7759/cureus.37307

**Published:** 2023-04-08

**Authors:** Vladimir Rubinshteyn, Vincent Giordano, Douglas Cohen, Johnathon LeBaron, Sujoy Menon, Christopher Demaree

**Affiliations:** 1 Trauma Surgery, Richmond University Medical Center, Staten Island, USA; 2 Trauma Services, Richmond University Medical Center, Staten Island, USA; 3 Neurosurgery, Richmond University Medical Center, Staten Island, USA; 4 Emergency Medicine, Richmond University Medical Center, Staten Island, USA; 5 Radiology, Richmond University Medical Center, Staten Island, USA; 6 Trauma Surgery, State University of New York Downstate Health Sciences University, Brooklyn, USA

**Keywords:** traumatic brain injury, injury mechanism, injury risk, sex differences, moderate/severe tbi, major trauma

## Abstract

Introduction

Traumatic brain injuries (TBIs) affect millions of patients each year, with more than 220,000 hospitalizations in 2019 and 64,000 deaths in 2020 alone. TBIs span a plethora of injuries including cerebral contusions and lacerations, axonal injuries, optic pathway disruptions, and skull fractures. Previous research has established that characteristics such as sex, mechanism of injury (MOI), and blood-thinning agents have some causal connections to a variety of cranial traumas.

Methods

This paper sought to analyze aggravating risk factors for various TBIs in the New York City borough of Staten Island. Data on eight predictive risk variables were collected at a level 1 trauma center from January 1, 2022, to December 31, 2022: MOI, seizure history, anticoagulant/antiplatelet therapy, alcoholism, age, biological sex, tested alcohol level, and body mass index (BMI). A multinomial logistic regression was estimated to generate risk ratios (RRs), and chi-squared tests were carried out to determine univariate associations.

Results

It was found that blood thinner use and sex were both significant predictors of various types of TBIs. Additionally, those not tested for alcohol, including pediatric patients, were less likely to suffer most forms of TBI, while BMI had a negligible relationship with any TBI class. The use of blood-thinning agents put patients at an increased risk of concussions (relative risk ratio [RRR]: 1.82; 95% confidence interval [CI]: 1.10-3.02) and undiagnosed intracranial injuries (RRR: 1.90; 95% CI: 1.08-3.34). Men were at higher risk of multiple cranial injuries than women (RRR: 3.62; 95% CI: 1.38-9.48), as well as physical traumas such as brain lacerations and hemorrhages (RRR: 2.81, 95% CI: 1.28-6.18). BMI was weakly correlated with undiagnosed cranial injuries (RRR: 1.04; 95% CI: 1.00-1.08) and slightly uncorrelated with physical traumas (RRR: 0.94; 95% CI: 0.88-1.00). Those not tested for alcohol were at far less risk of multiple TBIs (RRR: 0.08; 95% CI: 0.01-0.66), concussions (RRR: 0.27; 95% CI: 0.11-0.71), and physical brain traumas (RRR: 0.33; 95% CI: 0.13-0.84). No parameter exhibited any statistical significance with skull fractures.

Conclusion

Particular risk factors for TBIs include biological sex and blood thinner use. Injury prevention efforts should be based on the category of TBI, with a particular focus on blood thinner users becoming concussive post-trauma. Attention should also be paid to men who engage in risky behavior such as binge drinking and crime sustaining more than one brain trauma or isolated brain bleeds. Therefore, improved hospital outreach for fall precautions in nursing homes and targeted interventions for at-risk men are vital for future projects.

## Introduction

Traumatic brain injuries (TBIs) span a wide array of different injuries based on the nature of a patient’s trauma, comorbidities, pre-existing conditions, age, and access to medical care. Generally, these injuries are caused by either blunt force trauma to the head or penetrating cranial injuries such as gunshot wounds (GSWs) and stabbings. Additionally, they may potentially alter the fundamental neurochemistry and structure of a person’s brain, with the possibility of long-term physical, mental, and emotional detriment. Furthermore, resulting medical complications may cause unplanned future hospitalizations and place burdensome healthcare expenses on affected families.

TBIs include diverse and often overlapping intracranial injuries. The most common types of TBIs include concussions and any mild intracranial injury resulting in a loss of consciousness (LOC). However, more severe injuries such as skull fractures, lacerations, and contusions of the cerebrum, and subdural bleeds are possible and strongly correlated to more high-energy mechanisms of injury (MOIs) such as falls from high elevations and GSWs. Neurosurgical interventions are undergirded by the severity of the TBI and may include hematoma evacuations, craniectomies, and the placement of intracranial pressure monitors to observe the course of the injury.

According to the Centers for Disease Control and Prevention (CDC), approximately 176 U.S. citizens died each day from TBIs and other germane injuries in 2020, with hundreds of thousands of hospitalizations in 2019 alone. Statistics indicate that certain groups are at a heightened risk of TBIs, with vulnerable members of society such as homeless veterans, domestic violence survivors, and rural patients facing a greater propensity of suffering TBIs and related traumas and facing enduring complications. In addition, traumatic falls are the leading cause of TBI-related hospitalizations, and suicides by firearm are the leading cause of TBI-related deaths in the US. Alarmingly, geriatric patients suffering traumatic falls account for around a third of all TBI-related hospital admissions [1].

The long-term effects of TBIs vary according to their severity. Mild and moderate TBIs such as concussions do not often result in mortality and typically require non-acute interventions such as neurological observations, abstention from physical exercise, and several days of bed rest. On the other hand, more severe TBIs such as skull vault fractures may lead to nerve cell decay, chronic nervous system injuries, mental impairment, and, in the most severe cases, diffuse axonal injury with resulting coma. Those with moderate-to-severe TBIs saw an average reduction in lifespan of 6.6 years. In the most extreme TBI cases, less than a third of patients were ever able to return to normal everyday life [2]. Apart from the chronic neurobiological and psychiatric effects of TBIs, some research indicates a causal relationship with increases in violent crime. One study found that children and adolescents with TBIs such as concussions were more likely than their peers to commit violent crimes, resist medical and behavioral treatments, and re-offend later in life [3].

Considerable attention has been paid by state and private actors to managing the acute and chronic effects of TBIs, with more research currently being carried out on particular risk factors for TBIs. Existing scholarship does identify a cadre of aggravating risk factors for TBIs. One study from Central Europe indicated an increased risk of moderate-to-severe TBIs in geriatric antiplatelet and anticoagulant users. Specifically, patients on vitamin-K antagonists were 1.89 times as likely to suffer a severe TBI relative to non-blood thinner users [4]. Research from Spain reinforces the risk associated with these medications, with a noticeable correlation with age. These patients were less likely to tolerate surgeries and therefore had worse outcomes overall [5]. Patients on anticoagulation and other blood-thinning therapies are unable to clot their blood effectively, leading to delayed intracranial bleeds and other neurological complications [6]. Interestingly, research from Michigan and Ohio shows a divergence in injury epidemiology between oral anticoagulants and antiplatelet agents, with oral anticoagulants such as warfarin putting patients at an increased risk of mortality and hospice need [7].

Other factors may aggravate the severity and duration of TBIs. There appears to be some correlation between biological sex and concussions, with men more likely than women to suffer from mild-to-moderate TBIs. Furthermore, past and recurrent histories of TBIs were also critical predictive factors [8]. Alcohol intoxication is arguably one of the most robust predictive risk factors for most types of TBIs. This relationship is multidimensional, with patients suffering from TBIs who consume alcohol post-trauma more likely to face recurrent TBIs, longer recovery horizons, and worse prognoses. Additionally, recent literature delineates that cranial traumas are correlated with moderate-to-severe alcohol abuse [9]. Sports such as football and ice hockey were also aggravating factors for concussions [10]. Interestingly, despite the association between the average male and concussions, female athletes sustained concussions more frequently irrespective of the sport. Yet, there is significant underreporting of concussions among male athletes, likely skewing the sex association in sports injuries [11,12]. Van Pelt et al. support the sports and sex relationship established in prior studies; from their research in U.S. service academies, it was found that female cadets were 2.02 times at greater risk of concussions overall compared to male cadets. Attention deficit hyperactivity disorder (ADHD) and prior history of headaches also put cadets at risk of concussions [13]. Yet, Farley et al. conclude that limited evidence supports the conclusion that female athletes are at an increased risk of general sports injuries, let alone cranial traumas [14]. In general, athletes with at least one past concussion were far more likely to sustain a future TBI in contact sports than their peers [15].

In this study, we sought to analyze which demographic, medical, and social factors place patients at heightened risk of various forms of TBI at a level 1 trauma center. Data on all trauma patients arriving at Richmond University Medical Center's (RUMC) Emergency Department between January 1, 2022, and December 31, 2022, were queried from our trauma registry. A multinomial logistic regression with six outcome classes was estimated with a significance level of α=0.05.

## Materials and methods

The aim of this study is to isolate exogenous variables that may predictably put patients at risk of a variety of cranial traumas. Due to the plethora of potential TBIs to study, this paper initially identified the following types of injuries to be classified: 1) cerebral contusions, hemorrhages, lacerations, and edemas; 2) fractures of the skull, including both vault and base; 3) mild TBIs such as concussions with and without LOC; and 4) diffuse and focal traumas, including herniations and compressions.

For this study, the 10th Revision of the International Classification of Diseases (ICD) was used to identify which diagnosis codes correspond to the various cranial traumas. All trauma injury codes start with either “S” or “T,” and all head injuries are categorized from S00 to S09. The code prefixes given in Table 1 were initially selected based on guidance from the National Institutes of Health and CDC.

**Table 1:** ICD-10 codes for TBIs. TBI, traumatic brain injury, ICD-10, International Classification of Diseases 10th Revision

TBI Class	ICD-10 Codes
Skull fractures	S02.0, S02.1
Unspecified and other skull and facial fractures	S02.8, S02.91
Injuries to optic pathways and cortexes	S04.02, S04.03, S04.04
Intracranial traumas including concussions and hemorrhages	S06
Crushing injuries to the skull	S07.1
Shaken baby syndrome (SBS)/abusive head trauma (AHT)	T74.4

Six classifications were created for this study. Injuries with codes S02.0, S02.1, S02.8, or S02.91 were categorized under the *Skull Fracture* class. Code S06.0 corresponds to concussions and was thus classified as *Concussion*. All other S06 codes, S06.1 through S06.A (excluding S06.9), correspond to hemorrhages, hematomas, lacerations, traumatic lesions, and compressions. These are all intracranial injuries that may be visible on computed tomography (CT) or magnetic resonance imaging (MRI) scans. Due to the small number of S06.1-S06.A injuries regularly treated at the study site, segmenting these codes into smaller categories was imprudent. Therefore, these were classified under the class *Physical Intracranial Injury*, as was the S07.1 code prefix. Code S06.9A is for unspecified intracranial injuries and was thus classified under the *Unspecified Intracranial Injury* class. Shaken baby syndrome/abusive head trauma (SBS/AHT)-induced TBIs may result in a myriad of different cranial traumas and therefore will be classified on a case-by-case basis. Patients whose injuries satisfy two or more classifications were categorized under *Multiple TBIs*. All other patients without TBIs were classified under No TBI. Codes S04.02, S04.03, and S04.04 are rarely seen at the study site and would present too few observations to warrant their own category. A multinomial logistic regression was estimated using the eight predictor variables displayed in Table 2.

**Table 2:** Theoretical exogenous variables. TBI, traumatic brain injury; ETOH, ethyl alcohol; ED, emergency department; BMI, body mass index

Variable	Description	Predicted Impact
x_1_	Blunt trauma (binary variable): yes, no	Strong correlation with all TBIs
x_2_	History of blood thinner use (binary variable): yes, no	Strong correlation with contusions/hematomas
x_3_	History of seizures/epilepsy (binary variable): yes, no	Modest correlation with all TBIs
x_4_	History of alcohol abuse (binary variable): yes, no	Strong correlation with all TBIs
x_5_	ETOH level at ED triage (polytomous): not tested, not detected, trace levels, beyond legal limit	Higher intoxication = more severe TBIs
x_6_	ED-measured BMI raw score	Unknown
x_7_	Age cohort (polytomous): pediatric (<15), youth (15 to 24), adult (25 to 64), geriatric (>65)	Strong pediatric correlation with concussions; strong geriatric correlation with brain bleeds
x_8_	Biological sex (binary variable): male, female	Strong correlation between concussions and men

Blunt traumas may result from geriatric falls, motor vehicle collisions, assaults, and sports injuries, and typically involve a higher level of energy to the whole body than a penetrating trauma. Therefore, it is predicted that blunt traumas will be strongly correlated with most TBIs. Additionally, reduced clotting resulting from blood-thinning agents may put patients at an increased risk of delayed TBIs such as subdural hemorrhages. Patients with a history of seizures may be more likely to sustain head strikes against dull surfaces, being unable to brace during falls in the way fully conscious patients can, thus leading to more severe TBIs. In addition to seizures, a history of alcohol abuse may be tethered to a history of falls, which would increase the likelihood of recurrent TBIs. Alcohol use during a traumatic event has also been linked to a greater propensity to sustain a TBI, as intoxicated patients lack the full coordination of their senses during the event and may be less likely to avoid traumatic injury. For biological sex, men are at a higher risk than women of suffering cranial traumas due to their engagement in riskier activities such as binge drinking. Children and the elderly are more likely to sustain cranial traumas from high-energy injuries due to more fragile bone and cranial structures and more comorbidities among older patients. Body mass index (BMI) is expected to track closely with the number of comorbidities such as cardiac problems and alcohol abuse. However, obesity may lead to less concentrated trauma to the head during injury, thus confounding the exact predicted relationship with TBIs.

Raw data were collected and then randomized using the trauma registry of RUMC, a level 1 adult and level 2 pediatric trauma center in the New York City borough of Staten Island. The data were confidentially collected by the Trauma Department, and a queried report was delivered to the study team. Data were collected on all verified trauma patients with at least one ICD-10 diagnosis from January 1, 2022, to December 31, 2022. This resulted in n=1,302 patients for the population. The following raw data elements were initially queried in the report: 1) random unique ID (added by the Trauma Department), 2) ICD-10 diagnosis code using a prefix of either S or T, 3) patient age at the time of visit, 4) mechanism of injury (i.e., motor vehicle accident, assault, GSW, etc.), 5) comorbidities/medical history (a full list of reported medical diagnoses per patient), 6) level of recent alcohol consumption, and 7) BMI.

All exogenous variables, except for BMI, were transformed into categorical binary or multinomial predictors of the endogenous outcomes for TBIs. Age was broken down into four categories: 1) pediatric (<15 years old), 2) youth (15 to 24 years old), 3) adult (25 to 64 years old), and 4) geriatric (>65 years old). Alcohol level was similarly segmented: 1) not tested (no ethyl alcohol [ETOH] screening order), 2) not detected (<11 mg/dL), 3) trace levels (11 to 79 mg/dL), and 4) beyond the legal limit (>79 mg/dL). Registry/medical record numbers, triage dates, and any other sensitive patient information were removed by the Trauma Department to protect patient privacy and ensure HIPAA compliance.

## Results

Initial descriptive statistics were calculated and organized in Table 3. 

**Table 3:** Descriptive endogenous statistics. TBI, traumatic brain injury

TBI Reported	Total Count	Percent of TBIs
No TBI	1,051	N/A
Concussion	85	33.86%
Multiple TBIs	37	14.74%
Physical intracranial injury	46	18.33%
Skull fracture	19	7.57%
Unspecified intracranial injury	64	25.50%

TBIs accounted for 19.28% of all diagnosed traumas at RUMC in 2022. Hence, one in every five RUMC patients will be diagnosed with cranial trauma. Concussions accounted for approximately a third of all reported TBIs at RUMC in 2022 or 6.53% of all patients. Thus, one in every 12 RUMC patients will be diagnosed with a concussion. To evaluate the relative risk associated with particular aggravating factors, this study conducted a multinomial logistic regression (*mlogit*) on all 1,302 observations with *No TBI* as the base outcome. Stata was used to import, clean, and analyze the data. The constant term was not forced to zero as this often skews estimates and creates model bias. Results for relative risk ratios (RRRs) were estimated for the *mlogit* model, which compares the risk of specific TBIs in some parameter classes relative to others. Variables were individually tested for significance using Wald tests, which produced the following statistics in Table 4.

**Table 4:** Initial Wald test results. ETOH, ethyl alcohol; ED BMI, emergency department body mass index

Variable	χ2 Test Statistic	p-value
Blunt trauma	3.97	0.553
Blood thinners	5.39	0.370
Seizures	5.46	0.363
Alcoholism	3.07	0.690
ETOH level	28.68	0.018
ED BMI	12.26	0.031
Age cohort	12.01	0.678
Sex	17.00	0.005

To produce the strongest *mlogit* model of TBI risks, only those variables significant at α=0.05 were kept. Initially, the variable for alcoholism was excluded with a p-value of 0.690, and the model was run again. The age cohort parameter failed with a p-value of 0.712 and was subsequently removed. The third iteration resulted in the blunt trauma variable's exclusion with a p-value of 0.512 and was excluded. In the fourth iteration, seizure history produced a p-value of 0.243 and was similarly removed. The remaining four exogenous variables of blood thinners, BMI, ETOH level, and sex were significant at the 95% confidence level. The following final Wald test results were obtained and organized in Table 5.

**Table 5:** Final Wald test results. ETOH, ethyl alcohol; ED BMI, emergency department body mass index

Variable	χ2 Test Statistic	p-value
Blood thinners	13.08	0.023
ETOH level	28.78	0.017
ED BMI	13.26	0.021
Sex	16.14	0.006

To test for model misspecification, a Wilks log-likelihood-ratio test was performed on the initial model. The test ran a constrained model with BMI and sex removed against the full unconstrained model. We assumed that the constrained model was nested within the full model and that including BMI and sex in the full unconstrained model would result in more accurate risk estimations relative to the nested model. This produced a test statistic of χ2=32.60 and a p-value of 0.001. Therefore, the constrained model was rejected in favor of the full unconstrained model.

As this study seeks to measure predictive risk factors for various TBIs, the *mlogit *model was run with RRRs as its coefficients. Table 6 displays the statistically significant risk ratios for the *Concussion*, *Multiple TBIs*, *Physical Intracranial Injury*, and *Unspecified Intracranial Injury* classes. No variable carried any statistical significance with the Skull Fracture class at the α=0.05 level, excluding it from the final analysis.

**Table 6:** Risk ratios for the significant parameters. TBI, traumatic brain injury; RRR, relative risk ratio; ED BMI, emergency department body mass index; ETOH, ethyl alcohol

Variable	TBI Class	RRR	Standard Errors	p-Value	95% CI
Blood thinners	Concussion	1.819	0.470	0.020	1.097-3.018
	Unspecified intracranial injury	1.897	0.546	0.026	1.079-3.337
ETOH level (not tested)	Concussion	0.273	0.133	0.007	0.105-0.707
	Multiple TBIs	0.080	0.086	0.019	0.010-0.662
	Physical intracranial injury	0.327	0.158	0.020	0.127-0.842
ED BMI	Physical intracranial injury	0.939	0.029	0.043	0.884-0.998
	Unspecified intracranial injury	1.038	0.019	0.035	1.002-1.076
Sex (male)	Multiple TBIs	3.617	1.778	0.009	1.380-9.478
	Physical intracranial injury	2.811	1.131	0.010	1.278-6.184

For concussions, those on anticoagulation and antiplatelet therapies such as warfarin, heparin, and clopidogrel were 1.82 times at an increased risk than those not taking blood thinners. However, those not tested for alcohol (including adult patients for whom no drug screening was performed) were less likely to sustain concussions than those tested without positive results or with trace levels. Those suffering multiple cranial traumas (e.g., both axonal injuries and skull fractures) were 3.62 times at greater risk if they were men compared to women, and those not tested for alcohol were at far less risk. Similarly, men were 2.81 times more at risk of sustaining a physical cranial trauma such as a cerebral hematoma or laceration than women. Higher BMIs correlated with a negligible reduction in physical trauma risk, while those not tested for alcohol were at reduced risk. Finally, blood thinner use resulted in 1.90 times increased risk of unspecified brain traumas, while higher BMIs had a negligibly augmented risk.

## Discussion

The results of the *mlogit* model buttress the literature review and the theoretical model it undergirds while offering some departures. Men were, on average, two to three times at an increased risk of sustaining cranial traumas compared to women, with sharp divergence in the *Multiple TBIs* class. Men were more at risk of physical traumas such as cerebrum bleeds and multiple injuries. This may be due in part to men being more likely to undertake risky activities in the borough such as crime, reckless driving, and physical assaults. Similar to what was found by studies cited in the literature review, sex did not have a cognizable association with concussions at the 95% confidence level. As concussions may be milder than the severe axonal injuries men are more at risk for, sex, therefore, did not present any traceable correlation in the data.

Risks associated with blood-thinning agents were supported by the analysis. Generally, the use of antiplatelets and anticoagulating agents put their users anywhere between 1.5 and 2.0 times at an increased risk of developing either a concussion or unspecified cranial trauma. What was surprising was the lack of significance with physical injuries such as hematomas. While association with lacerations may not have much theoretical support, some effect on contusions and bleeds was expected. A potential explanation is that most blood thinner users suffered traumatic falls at low speed and low energy. Hence, their probability of sustaining moderate or severe TBIs was less than younger patients who experienced more high-energy injury mechanisms such as motorcycle crashes.

The lack of statistical significance for any variable class under *Skull Fractures* was indeed surprising. However, the limited collection of diagnosed traumatic skull fractures across the entire year may partially explain this phenomenon. In total, only 19 isolated vault and basilar skull fractures were reported in 2022, which represented 7.57% of all TBI diagnoses. For the remaining 232 TBI patients, 85 reported concussions, 64 had unspecified cranial traumas, 46 sustained physical cranial traumas, and 37 patients were diagnosed with more than one TBI. In addition, the *Multiple TBIs* category does contain some patients with both skull fractures and other cranial traumas. Yet even assuming that 5%-10% of patients in this class sustained skull fractures, the number may still be too small to lead to any significance at α=0.05.

Patients not tested for alcohol were less likely to sustain a TBI across all trauma categories. On average, eligible adults not tested for ETOH had only a fifth of the risk as those who were tested. ED provider judgment may be a causal factor in this inverse relationship. Attending physicians who encounter TBI patients were more likely to test for drugs and alcohol, as these factors may aggravate potential TBI-related complications and hamper immediate surgical interventions. On the other hand, patients presenting with milder non-TBI injuries such as scalp edemas and unspecified extremity pain may not warrant as frequent or consistent screening.

BMI had a noticeably minuscule effect on both physical cranial traumas and unspecified cranial traumas. On average, more obese patients had a risk ratio of 0.989 for TBIs at the 95% confidence interval. It was initially predicted that those with higher body masses would correlate with more comorbidities. As more preexisting conditions may put patients at an increased risk of TBI and TBI-related complications, some relationship was expected. However, given the effectively neutral effect of BMI on any of the TBI classes, this assumption seems to have been invalidated.

Limitations

We acknowledge several limitations to our study design and analysis. Our study made use of a polytomous regression model with six TBI outcome classes. While our model did confirm previous literature regarding the risks of blood-thinning agents and riskier activities among men, its overreliance on binary and multinomial parameters may have led to bias in the estimated risk ratios. The accepted convention for the number of binary exogenous parameters is k−1, where k is the number of endogenous outcome classes, and our model contains six outcome values and five binary predictors. Nevertheless, only one of the remaining three variables was continuous (ED BMI). The remaining two parameters, ETOH level and age cohort, each contained four classes. While our maintenance of the k−1 rule reduces the probability of a "dummy variable trap," more continuous variables could have been incorporated. A second limitation is the lack of statistical significance of any parameter with the *Skull Fracture* category. While skull fractures constituted less than 8% of all reported TBIs and approximately 1.5% of all trauma diagnoses in 2022, they are nonetheless severe TBIs that warrant further investigation. Estimating prediction parameters for skull fractures would help inform injury prevention and community health outreach initiatives.

Another shortcoming is the retrospective nature of the study. A prospective study would have followed the course of cranial traumas in real time, allowing for more precise risk ratios to be estimated. However, due to the volatile and unpredictable nature of TBIs, such as potential comas and mortalities, maintaining a consistent sample size during the course of the study may have proven improbable. Nevertheless, great care was taken to build a statistical model based on a rigorous literature review and a priori analysis.

## Conclusions

TBIs affect millions of patients and cause tens of thousands of deaths each year. This study sought to identify those risk factors with the strongest correlations with various cranial trauma classifications. A multinomial logistic regression was run on all trauma patients presenting to Richmond University Medical Center in 2022. It was found that blood thinner use and biological sex were the strongest predictors of cranial trauma. Not testing for alcohol was associated with reduced risk for TBI due to a correlation with non-TBI injuries and low-energy injury mechanisms. Future injury prevention efforts should be concentrated on geriatric blood thinner users at home and in skilled nursing facilities, as well as men who engage in risky behaviors.
